# The seven virtues of *Samurai*: Revisiting a classic through computational language analysis

**DOI:** 10.1093/pnasnexus/pgag314

**Published:** 2026-09-17

**Authors:** Ryota Takano, Minoru Karasawa

**Affiliations:** Department of Cognitive and Psychological Sciences, Graduate School of Informatics, Nagoya University, Furo-cho, Chikusa-ku, Nagoya 464-8601, Japan; Education and Research Center for Future Value Creation, Graduate School of Informatics, Nagoya University, Furo-cho, Chikusa-ku, Nagoya 464-8601, Japan

**Keywords:** culture, morality, historical psychology, natural language processing

## Abstract

The longstanding dominance of values rooted in Western, Educated, Industrialized, Rich, and Democratic (WEIRD) societies has increasingly come under scrutiny across the social sciences. This has renewed interest in how value systems cultivated in less-WEIRD contexts are articulated and widely received by WEIRD audiences. Recent advances in natural language processing make it possible to examine how such values are articulated in classical texts, using psychologically grounded analytical frameworks. The present study focuses on *Bushido: The Soul of Japan*, a widely read classic written by Inazo Nitobe to introduce Japanese ethical ideals to Western audiences. Using contextualized construct representation (CCR), a theory-driven computational approach to psychological text analysis, we examined how moral concerns are structured and expressed in *Bushido* relative to other texts related to Japanese culture, Christianity, and the Bible. Results showed that *Bushido* exhibits a distinct and structured moral profile that corresponds to psychologically defined moral dimensions. Moreover, among the Japanese culture-related texts, greater similarity to *Bushido* in moral profile was associated with broader reception in Western contexts, as indexed by independent indicators of publication, translation, and scholarly attention. Analyses of virtue-level structure further identified *Meiyo* (名誉; honor) as the conceptual core of *Bushido*'s moral framework, a form of honor distinct from Western pride-based conceptions. Together, these findings suggest that *Bushido* conveys moral values rooted in Japanese cultural traditions in forms that are psychologically intelligible to Western readers, illustrating how less-WEIRD cultural values can be communicated across cultural boundaries without losing their distinctive meaning.

Significance statement Why are some cultural traditions taken up beyond their original cultural contexts? We analyzed *Bushido: The Soul of Japan*, a widely read classic that introduced samurai ethics to Western audiences, using a theory-driven computational language analysis grounded in psychology. We found that *Bushido* organizes culturally rooted moral values into a structured profile that corresponds to psychologically defined moral dimensions, while preserving culturally distinctive features. Importantly, among Japanese culture-related texts, greater similarity to *Bushido* in moral structure was associated with broader reception in Western contexts. Together, these findings illustrate how classical less-WEIRD (Western, Educated, Industrialized, Rich, and Democratic) texts can convey culturally specific value systems in forms that are accessible to WEIRD audiences, shedding light on how cultural traditions travel across societies without losing their core meanings.

## Introduction

For centuries, values and ideologies from Western, Educated, Industrialized, Rich, and Democratic (WEIRD) societies have often been regarded as a dominant point of reference broadly applicable to diverse sociocultural systems. However, in recent years, this view has come under greater scrutiny, with growing criticism of the WEIRD model as a normative standard (1–5). This shift reflects an expanding emphasis on global and culturally diverse perspectives in academic disciplines such as psychology and related social sciences (4, 6), alongside broader social and political transformations in many parts of the world (7–9). These trends have prompted a reexamination of the WEIRD model's dominance and a renewed interest in value systems rooted in less-WEIRD societies. To address these social and scholarly developments, two key questions emerge: How can less-WEIRD perspectives and voices be communicated in ways that are intelligible to WEIRD audiences and can facilitate broader reception? More importantly, how can such processes of articulation and reception be assessed quantitatively? Recent advances in natural language processing techniques make it possible to examine classical less-WEIRD texts through contextual language models. Specifically, we investigate how *Bushido: The Soul of Japan*, a Japanese classic originally written in English, articulates traditional moral values in forms that resonate with Western audiences.

Our focus on a Japanese classic is grounded in Japan's distinctive historical and cultural position at the crossroads of WEIRD and less-WEIRD worlds (10, 11). After avoiding colonization and embracing modernization in the late 19th century, Japan rapidly developed into an industrialized nation that adopted Western systems while preserving its traditional values and retaining global cultural influence (12). This unique trajectory of modernization illustrates how cultural identity can evolve through selective integration rather than full assimilation into WEIRD norms.

One key work reflecting the cultural ethos during this period of drastic change is *Bushido: The Soul of Japan* (hereafter “*Bushido*”), originally written in English by Inazo Nitobe, who was born into a samurai family and later converted to Protestantism (13). *Bushido* was first published in 1899, at the height of Japan's Meiji-era transformations, a period marked by rapid political reform, cultural reconfiguration, and intensified engagement with Western powers, as Japan sought to redefine its national identity on the global stage. In this context, Nitobe explicitly sought to articulate the moral foundations of the samurai code for Western readers, countering the widespread assumption that Japan lacked a coherent moral system in the absence of Christianity. To this end, he deliberately framed *Bushido* through analogies to Western chivalry and Christian ethics, employing rhetorical strategies familiar to Western readers.

While Nitobe framed *Bushido* in ways that aligned with Western moral and rhetorical frames, he also systematically organized moral concepts that were already present in Japanese ethical thought, including samurai ethics, as well as traditions influenced by Confucianism and Buddhism, and presented them in *Bushido* as a coherent framework consisting of seven core virtues: *Gi* (義; rectitude), *Yu* (勇; courage), *Jin* (仁; benevolence), *Rei* (礼; politeness), *Makoto* (誠; veracity), *Meiyo* (名誉; honor), and *Chugi* (忠義; loyalty) (for brief explanations of these virtues, see Table 1). He argues that these values constitute the moral backbone of the samurai class and Japanese society. Against the backdrop of Nitobe's attempt to articulate Japanese moral values across cultural boundaries, the book became an international bestseller and played a significant role in shaping Japan's global image. Famously known as one of the US President Theodore Roosevelt's favorite books (14), it remains accessible today through various sources, most notably included in Project Gutenberg, the oldest digital library.

**Table 1:** Brief explanations of the seven virtues in *Bushido*.

Virtue	Explanation
*Gi* (義; rectitude)	Unwavering moral judgment based on reason; the foundation of just action and ethical duty.
*Yu* (勇; courage)	The virtue of acting with moral resolve and fearlessness, marked by calmness under pressure and the courage to do what is right.
*Jin* (仁; benevolence)	Compassionate love and empathy for others. True benevolence in *Bushido* balanced tenderness with justice and the power to protect.
*Rei* (礼; politeness)	Politeness as the outward expression of sympathy and respect for others. Beyond formal etiquette, it served as a spiritual discipline that cultivated inner harmony and moral self-restraint.
*Makoto* (誠; veracity)	Truthfulness signified inner strength and integrity. A samurai's word was binding without oath or contract. Lies were seen as cowardice, while sincerity, uniting word and being, was regarded as a near-sacred virtue.
*Meiyo* (名誉; honor)	Honor for the samurai meant an acute sense of personal dignity and moral worth. Rooted in family pride and shame-consciousness, it shaped behavior from childhood. True honor demanded patience, restraint, and self-discipline. Fame, not wealth, was the highest goal, and many samurai preferred death over disgrace.
*Chugi* (忠義; loyalty)	Absolute devotion to one's lord, even above family. True loyalty included honest counsel, not blind obedience.

Moral values, such as those articulated in *Bushido*, not only convey conceptions of what is good and how one ought to live but also serve as collectively shared frameworks within a society. Their shared nature entails a dynamic process of legitimization through mutual understanding and endorsement, which reinforces these values at the collective level over time (15–18). Given this communicative function and the unique historical–cultural context, analyzing the moral and psychological characteristics of a cultural text such as *Bushido* provides an opportunity to examine why certain value systems may resonate beyond their original sociocultural context. Rooted in traditional less-WEIRD values yet compelling to modern WEIRD readers, *Bushido* offers a particularly informative case for examining how moral values are expressed in ways that facilitate cross-cultural and historical transmission.

Guided by these considerations, the present study examines how moral concerns are structured and articulated in *Bushido*. To contextualize its moral profile, we compared *Bushido* with three reference corpora compiled from Project Gutenberg: Christianity-related texts, Bible-related texts, and other Japanese culture-related texts reflecting traditional cultural values. These corpora were treated as analytical benchmarks instead of direct competitors. The two Christian corpora were included as reference points because, like *Bushido*, they have historically been regarded as representative articulations of institutionalized elite moral thought within their respective cultural contexts (19, 20), providing a structured language through which broader cultural values are expressed.

To examine these texts within a common analytical framework, we employed contextualized construct representation (CCR), a recently developed method for psychological text analysis that combines theory-driven insights with representations derived from contextual language models (3, 21–24). CCR quantifies the extent to which a target text expresses specific moral-psychological constructs by computing semantic similarity between the text and items from validated psychometric questionnaires. Previous research has demonstrated that CCR can effectively capture psychological constructs in naturalistic texts, including historical documents from diverse cultural traditions (3, 21, 23–25).

As a common descriptive framework for CCR analysis, we incorporated the Moral Foundations Questionnaire-2 (MFQ-2), a widely used instrument grounded in Moral Foundations Theory (MFT) that captures multidimensional patterns of moral concern and has been extensively applied in cross-cultural research (26–29). The most recent version, developed by Atari et al., has demonstrated robust cross-cultural measurement invariance and validity and has also been employed in studies using the CCR approach (3, 21, 29). Accordingly, the MFQ-2 provides a standardized descriptive framework for characterizing how moral concerns are articulated across texts. In this sense, MFQ-2-based CCR scores reflect the extent to which a text articulates moral concerns in ways that correspond to psychologically defined dimensions of moral judgment, indexing the clarity and structure with which such concerns are expressed in language.

Based on this framework, we tested the following expectations: First, given its explicit intention to convey Japanese moral values to Western readers, we expected *Bushido* to exhibit a distinct and structured moral profile, as indexed by MFQ-2-based CCR scores, relative to Christianity-related, Bible-related, and other Japanese culture-related corpora. Second, we explored whether this moral structure was associated with broader reception in Western contexts. Finally, by analyzing the seven virtues described in *Bushido*, we examined how moral concerns were internally organized within *Bushido* itself.

## Results

### Moral profiles of *Bushido* relative to comparison corpora

We first compared the MFQ-2-derived CCR scores of *Bushido* with those of three comparison corpora comprising 225 Christianity-related, 466 Bible-related, and 30 Japanese culture-related documents (Fig. 1; see Methods and Supplementary information). A linear mixed-effects model revealed a significant interaction between document type (*Bushido*, Christianity-related, Bible-related, and Japanese culture-related) and MFQ-2 foundation, *F* (15, 25235.0) = 79.73, *P* < 0.001, indicating that the pattern of moral concern across the six MFQ-2 foundations differed systematically across the four document types (Table S1). Follow-up pairwise models further showed that *Bushido* exhibited a distinct overall moral profile relative to each comparison corpus (*F*s > 3.03, *p*s < 0.010; Table S1). *Bushido* showed the highest estimated marginal means across all MFQ-2 foundations, with differences from the comparison corpora ranging from 0.025 to 0.079 points; the clearest differences were observed for Authority, with corresponding gaps ranging from 0.054 to 0.079 (Fig. 1; Table S2). In foundation-specific planned contrasts using Dunnett's method, *Bushido* scored significantly higher than Bible-related and Japanese culture-related documents on Authority, and significantly higher than Japanese culture-related documents on Care (all adjusted *p*s < 0.025), whereas the remaining contrasts showed the same direction but did not reach significance after correction for multiple comparisons (Table S2).

**Figure 1:**
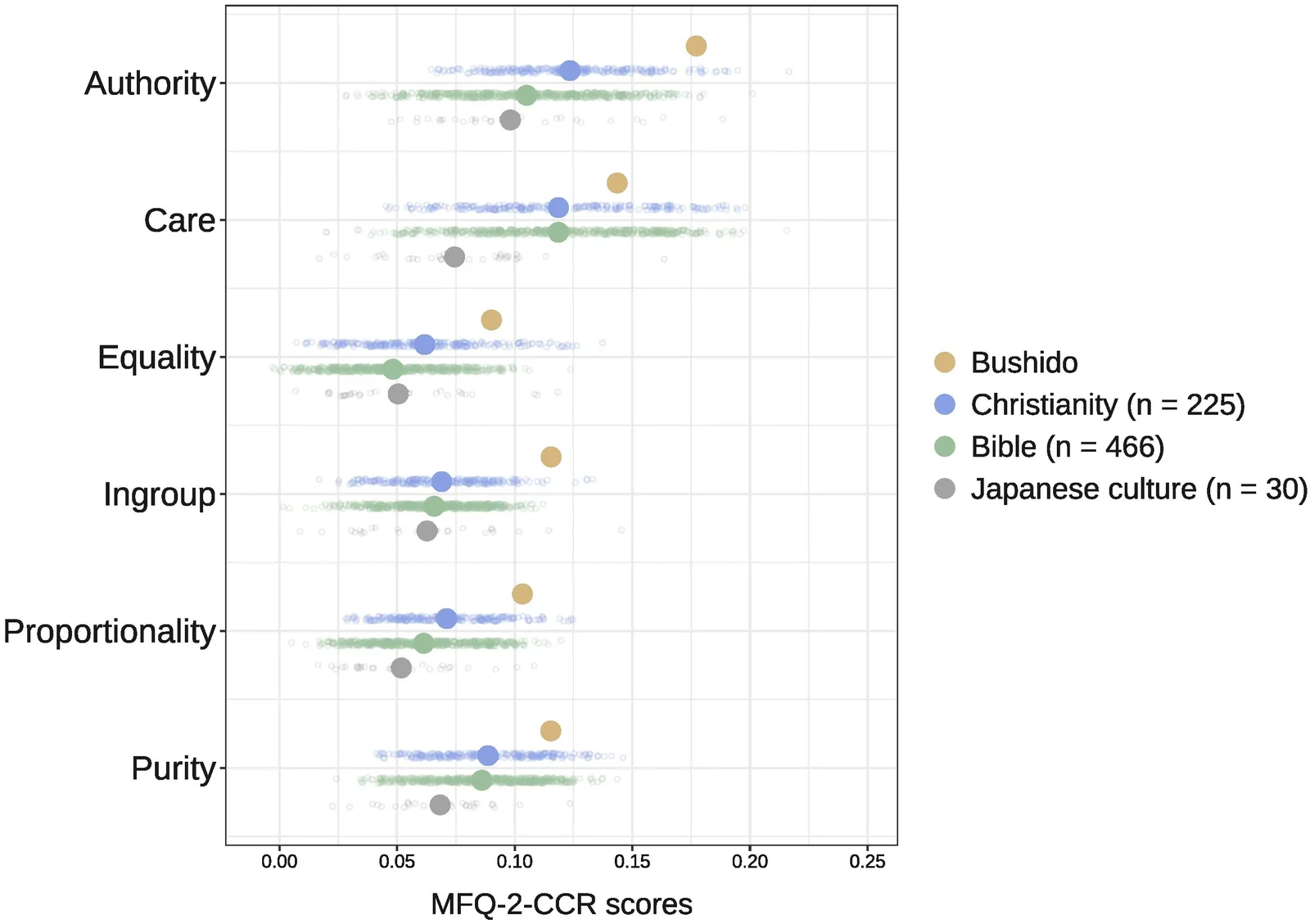
Moral profiles of *Bushido* and comparison corpora. Comparisons of MFQ-2-based CCR scores between *Bushido* and the Christianity-related, Bible-related, and Japanese culture-related corpora. The small symbols represent individual documents, whereas large symbols indicate estimated marginal means from the linear mixed-effects model for each document type.

Taken together, these findings suggest that *Bushido* exhibits a distinct and structured moral profile that corresponds to psychologically defined moral dimensions, with the clearest evidence observed for dimensions related to authority and social order. Notably, this overall pattern remained when the analysis was restricted to comparison documents authored by individuals whose lifespans overlapped with Nitobe's (1862–1933), suggesting that the distinctiveness of *Bushido*'s moral profile is not solely attributable to historical variations in writing style or content (see the Robustness checks section; Tables S3 and S4).

To illustrate how *Bushido* semantically aligns with moral-psychological constructs, we present representative sentences from the text that showed relatively high CCR scores across the MFQ-2 dimensions (Table 2). These examples offer qualitative insights into how moral constructs are embedded in the language of *Bushido*.

**Table 2:** Representative sentences from *Bushido* with high CCR scores across moral foundations.

Virtue	Sentence	Foundation	CCR score
*Meiyo* (名誉)	Said this sage, “’Tis in every man's mind to love honor: but little doth he dream that what is truly honorable lies within himself and not anywhere else.	Authority	0.42
*Jin* (仁)	The cultivation of tender feelings breeds considerate regard for the sufferings of others.	Care	0.56
*Makoto* (誠)	The separation of power and riches kept the distribution of the latter more nearly equable.	Equality	0.39
*Chugi* (忠義)	Similarly, Loyalty as we conceive it may find few admirers elsewhere, not because our conception is wrong, but because it is, I am afraid, forgotten, and also because we carry it to a degree not reached in any other country.	Ingroup	0.35
*Jin* (仁)	“Virtue is the root, and wealth an outcome.”	Proportionality	0.33
*Rei* (礼)	We may reverently say, politeness “suffereth long, and is kind; envieth not, vaunteth not itself, is not puffed up; doth not behave itself unseemly, seeketh not her own, is not easily provoked, taketh not account of evil.”	Purity	0.25

Each row represents a sample sentence aligned with a particular Moral Foundation Questionnaire-2 dimension, along with the associated virtue and CCR score.

### Moral profile similarity to *Bushido* and western reception

We examined the extent to which Japanese culture-related documents, including *Bushido*, have been received in Western contexts, and how such reception is associated with moral profile similarity to *Bushido*. First, we computed a western reception index (WRI) for each Japanese culture-related document, defined as a composite of the number of English-language editions, number of translations into non-English Western European languages, and citation counts indexed by Google Scholar, restricted to English-language sources (Cronbach's *α* = 0.78; see Methods for details (30–33)). The results showed that *Bushido* ranked second highest among the Japanese culture-related documents in terms of WRI (Fig. 2a), indicating that *Bushido* has been widely received in Western contexts as a work reflecting Japanese cultural values.

**Figure 2:**
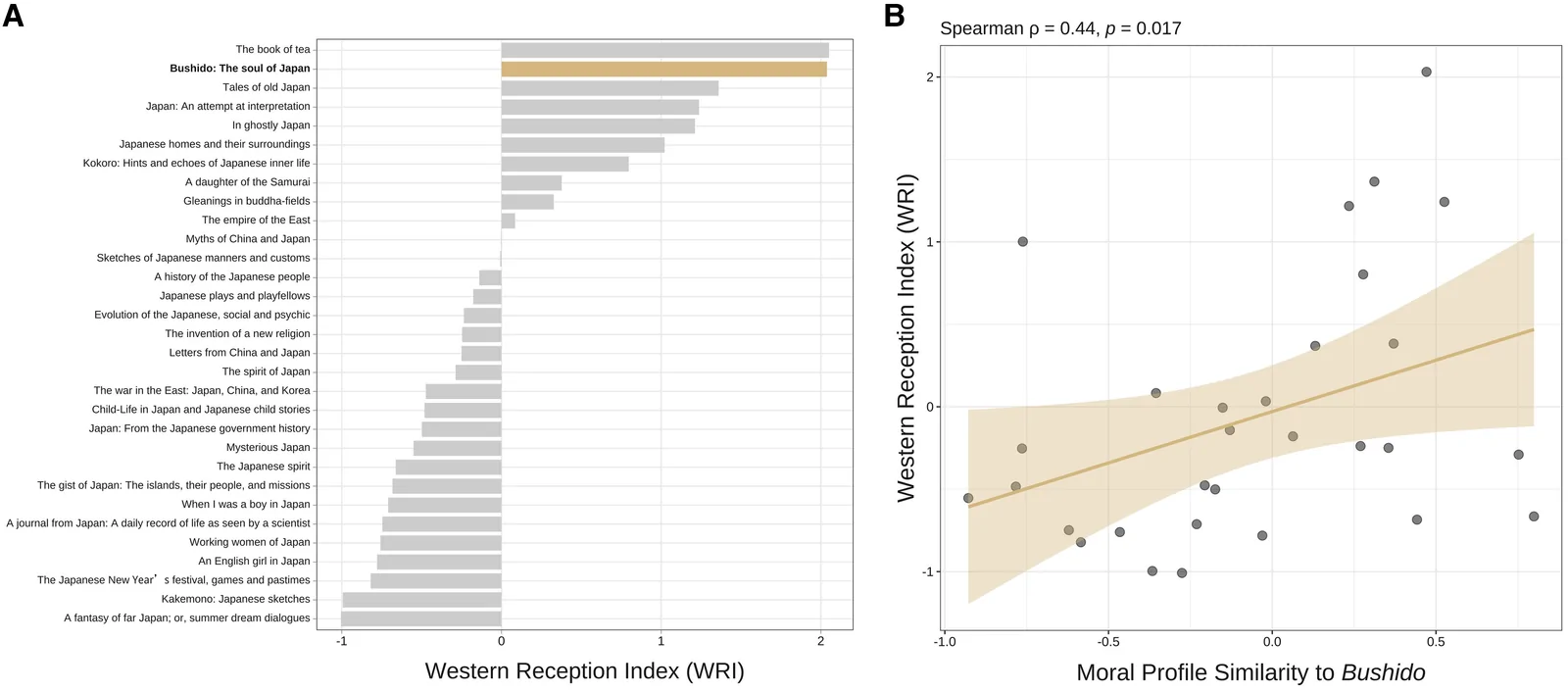
Western reception of Japanese culture-related documents and its association with moral profile similarity to *Bushido.* a) WRI for Japanese culture-related documents. The WRI was computed only for Japanese culture-related documents and represents a composite measure based on the number of English-language editions, number of translations into five non-English Western European languages, and citation counts indexed by Google Scholar, restricted to English-language sources. b) Association between moral profile similarity to *Bushido* and Western reception among Japanese culture-related documents, excluding *Bushido* itself. Moral profile similarity was quantified as the correlation between the MFQ-2-based CCR scores of each Japanese culture-related document and those of *Bushido*. The solid line represents the linear regression fit, and the shaded area indicates the 95% CI.

Next, we quantified moral profile similarity to *Bushido*, defined as the degree to which a document's relative profile of moral concerns across MFQ-2 foundations resembled that of *Bushido*, by computing correlations between MFQ-2-based CCR scores of each Japanese culture-related document and those of *Bushido*, and examined its association with WRI. Note that this correlation analysis was conducted among Japanese culture-related texts excluding *Bushido* itself. The results revealed a significant positive relationship between moral profile similarity to *Bushido* and Western reception (Fig. 2b; Spearman's *ρ* = 0.44, *P* = 0.017). This pattern suggests that Japanese cultural documents whose moral structures are more similar to that of *Bushido* have tended to be more widely received in Western contexts. Notably, additional robustness analyses, including correlations with individual WRI components, leave-one-out correlation analyses, and partial correlations controlling for publication year, yielded comparable effect sizes, and the substantive conclusions remained unchanged (see the Robustness checks section).

### Moral profiles across the seven virtues of *Bushido*

To further elucidate *Bushido*'s moral structure, we examined the moral profiles of its seven virtues using MFQ-2-based CCR analyses. Because each virtue is discussed in a separate chapter, the text could be segmented accordingly, allowing CCR scores to be computed for sentences within each chapter. A linear mixed-effects model showed significant main effects of virtue type and moral foundation and, importantly, a significant interaction between them (*F* (30, 15,995.0) = 24.83, *P* < 0.001), indicating that the seven virtues differed not simply in overall magnitude but in their patterns across the six MFQ-2 foundations (Fig. 3a; Table S5). Rather than conforming to a single common ranking across foundations, the virtues exhibited differentiated profiles of relative emphasis, suggesting that *Bushido*'s moral-psychological structure is organized through differentiated virtue profiles (see also Table S6). Descriptive summaries of the remaining, nonvirtue chapters of *Bushido* were broadly consistent with *Bushido*'s overall moral profile. Although their CCR scores were generally lower than those of the virtue-focused chapters, their moral foundation profiles showed a similar broad pattern, with relatively elevated Authority and comparatively lower Equality across most chapters, together with chapter-specific variation in emphasis (Table S7).

**Figure 3:**
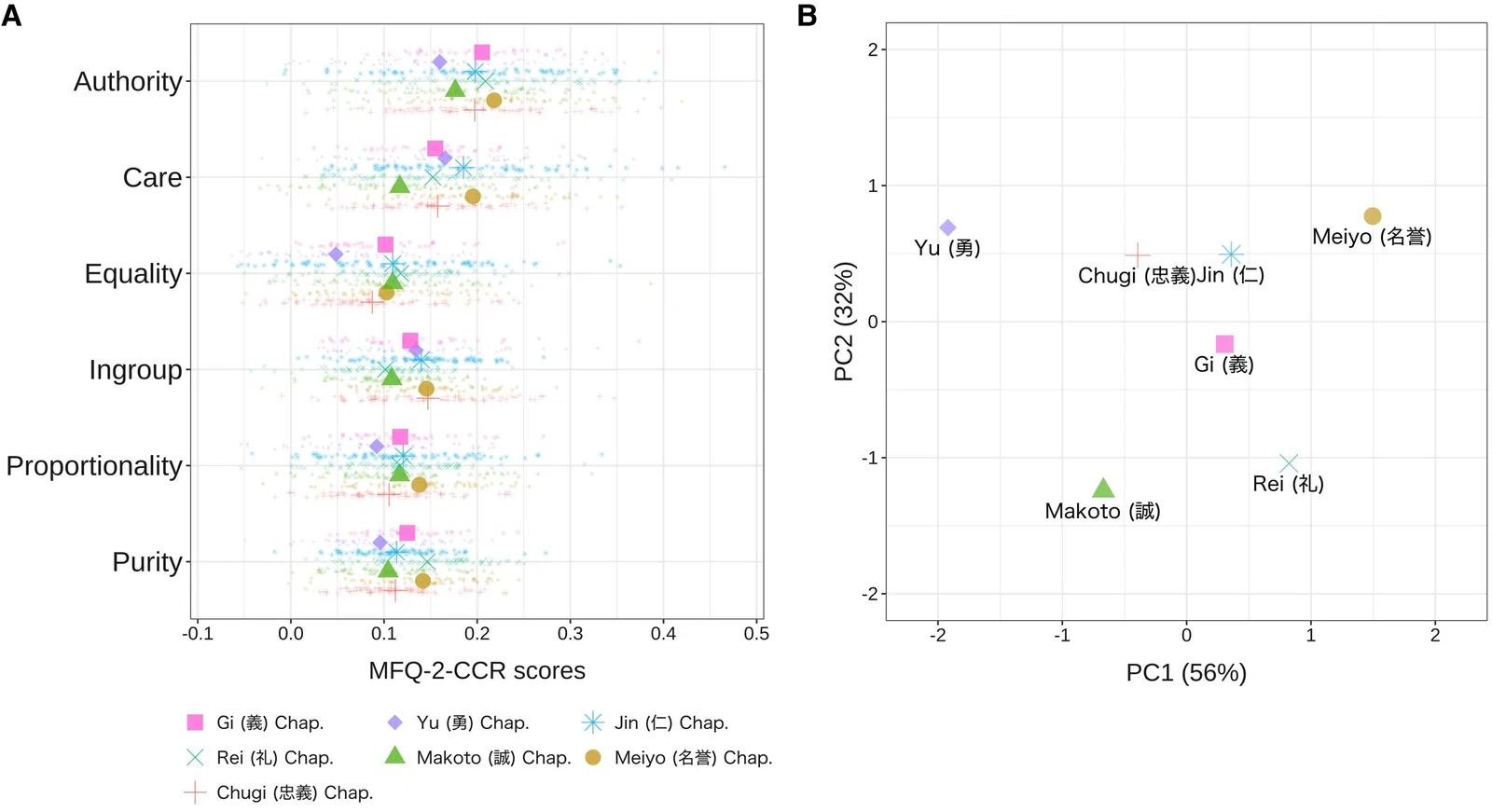
Moral profiles of Bushido’s seven virtues. a) MFQ-2-based CCR scores for the seven virtues of *Bushido*. The small symbols represent individual sentences from each virtue-specific chapter, whereas large symbols indicate the mean CCR scores for each virtue within each moral foundation. b) MDS configuration of the seven virtues of *Bushido* based on MFQ-2-derived CCR scores. Axes are interpreted with reference to PCA. PC1 is strongly associated with Authority, Equality, Proportionality, and Purity, whereas PC2 is associated with Care and Ingroup. Values in parentheses indicate the proportion of variance explained by PC1 and PC2 in the PCA. The seven virtues are *Gi* (義; rectitude), *Yu* (勇; courage), *Jin* (仁; benevolence), *Rei* (礼; politeness), *Makoto* (誠; veracity), *Meiyo* (名誉; honor), and *Chugi* (忠義; loyalty).

To characterize the latent organization of these virtue profiles, we applied multidimensional scaling (MDS) based on a dissimilarity matrix derived from the 7 × 6 matrix of mean MFQ-2-based CCR scores across virtues and foundations. We interpreted the axes based on dimensions identified through principal component analysis (PCA) (Fig. 3b). The first principal component (PC1) was positively associated with Authority, Equality, Proportionality, and Purity, reflecting a comprehensive moral orientation expressed in *Bushido* (Authority: *r* = 0.96, *P* < 0.001; Care: *r* = 0.44, *P* = 0.329; Equality: *r* = 0.78, *P* = 0.041; Ingroup: *r* = 0.02, *P* = 0.969; Proportionality: *r* = 0.91, *P* = 0.004; Purity: *r* = 0.90, *P* = 0.005). The second component (PC2), representing the variance *unaccounted for* by PC1, was positively correlated with Care and Ingroup (Authority: *r* = 0.03, *P* = 0.948; Care: *r* = 0.82, *P* = 0.023; Equality: *r* = −0.56, *P* = 0.191; Ingroup: *r* = 0.94, *P* = 0.001; Proportionality: *r* = −0.07, *P* = 0.887; Purity: *r* = −0.17, *P* = 0.720). With respect to the positioning of individual virtues, *Meiyo* (名誉), *Yu* (勇), *Jin* (仁), and *Chugi* (忠義) were positioned relatively high on the PC2-interpreted dimension, with *Meiyo* uniquely positioned high on both the PC1- and PC2-interpreted dimensions. This pattern suggests that *Meiyo* plays a central role in the moral structure articulated in *Bushido*.

### Robustness checks

To assess the reliability of the pretrained model, namely, “all-mpnet-base-v2” used in the main statistical analysis, we calculated the intraclass correlation coefficients (ICCs) between the CCR scores obtained from this model and those derived from three additional high-performing general-purpose models (i.e. “all-distilroberta-v1,” “all-MiniLM-L12-v2,” and “all-MiniLM-L6-v2”). The results indicated sufficient intermodel reliability (ICC = 0.868, 95% CI = [0.866, 0.870]), suggesting that our results remain consistent regardless of the specific pretrained model used.

As a complementary validation check of the CCR approach, we compared it with a representative psychological text analysis method: the dictionary-based approach. Specifically, we used the moral foundations dictionary (MFD) (34, 35) to compute dictionary-based scores using word frequency for each foundation and then calculated their correlation with CCR scores. The MFD categorizes words into those reflecting morally righteous actions and those associated with moral violations. Additionally, because the MFD follows the earlier five-foundation MFT framework, in which Fairness combines concerns that MFQ-2 separates into Equality and Proportionality, we averaged the CCR scores for Equality and Proportionality. We used data from all documents for this analysis. The results showed significantly positive correlations between CCR and dictionary scores for morally righteous words across all examined moral foundations (Authority: *r* = 0.10, 95% CI [0.02, 0.17]; Care: *r* = 0.59, 95% CI [0.54, 0.63]; Fairness: *r* = 0.38, 95% CI [0.32, 0.44]; Ingroup: *r* = 0.14, 95% CI [0.06, 0.21]; Purity: *r* = 0.51, 95% CI [0.45, 0.56]) and words representing moral violations (Authority: *r* = 0.12, 95% CI [0.05, 0.20]; Care: *r* = 0.23, 95% CI [0.16, 0.30]; Fairness: *r* = 0.18, 95% CI [0.11, 0.25]; Ingroup: *r* = 0.09, 95% CI [0.02, 0.16]; Purity: *r* = 0.45, 95% CI [0.39, 0.50]). Overall, these results suggest that CCR captures both morally “right” and “wrong” aspects of MFT-related content, thereby providing supportive evidence for the validity of the CCR approach.

To examine whether the observed effects could be attributed to historical differences in textual style or content, we re-ran the linear mixed-effects models using only documents authored by individuals whose lifespans overlapped with Nitobe's (1862–1933). The overall pattern remained similar in this contemporaneous subset of documents (160 Christianity-related, 274 Bible-related, and 27 Japanese culture-related documents): document type, moral foundation, and their interaction all remained significant, and *Bushido* continued to show the highest estimated marginal means across the six foundations, with the clearest differences observed for Authority (see Tables S3 and S4 for details). These findings suggest that the distinctiveness of *Bushido*'s moral profile is not solely attributable to historical-period differences in writing style or content.

We conducted three additional analyses to assess the robustness of the association between Western reception and moral profile similarity to *Bushido*. First, to examine whether the observed association was driven by any single component of the WRI, we separately examined the relationships between moral profile similarity to *Bushido* and each WRI component (i.e. the number of English-language editions, number of translations into five non-English Western European languages, and citation counts indexed by Google Scholar). The results showed that the correlations were comparable in magnitude across all WRI components (English editions: *ρ* = 0.35, Western translations: *ρ* = 0.37, Citations: *ρ* = 0.32), indicating that the observed association was not driven by any single indicator. Second, to examine whether the observed correlation was driven by any specific document, we performed a leave-one-out correlation analysis in which each Japanese culture-related document was sequentially excluded. The positive association remained stable across all iterations (*ρ* range = 0.39−0.52; median *ρ* = 0.43), indicating that the observed relationship was not driven by any single document. Third, to examine whether the observed association was attributable to publication period, we conducted a partial Spearman correlation controlling for the year of the first English edition. The positive association between moral profile similarity to *Bushido* and WRI remained significant after controlling for publication year (*ρ* = 0.42, *P* = 0.025).

## Discussion

In light of increasing global reflection on the limitations of WEIRD-centered perspectives in the social sciences, there has been growing interest in understanding how values cultivated in less-WEIRD contexts are articulated and communicated to audiences beyond their original sociocultural settings (3–5). *Bushido: The Soul of Japan*, written by a Japanese author for a Western readership at the turn of the 20th century, provides a particularly informative case for examining this issue. Building on this perspective, the present study examined how traditional moral values are structured and expressed in *Bushido*, how its moral profile is situated relative to other religious and cultural corpora, and how moral profile similarity to *Bushido* relates to the Western reception of other Japanese culture-related texts.

The CCR analyses showed that *Bushido* exhibited a distinct overall moral profile relative to the Christianity-related, Bible-related, and Japanese culture-related corpora. Across all six MFQ-2 foundations, *Bushido* showed the highest estimated marginal means, although the magnitude of these differences varied across foundations. The clearest contrasts emerged for the Authority foundation, which captures concerns with social hierarchy and order. Together, these findings suggest that *Bushido* articulates moral values deeply rooted in Japanese cultural traditions through a structured moral profile that corresponds to psychologically defined dimensions of moral judgment. The relatively elevated scores on Authority are also consistent with Nitobe's argument that Japanese morality stems from the feudal-era ethical principles of the samurai class (13).

A further important finding is that *Bushido* exhibited relatively high levels of reception within Western contexts among Japanese culture-related documents included in Project Gutenberg. More importantly, documents whose moral structures were more similar to that of *Bushido* also tended to exhibit higher levels of Western reception. Taken together, these findings suggest that the cross-cultural reception of *Bushido* may have been facilitated not only by the content of its moral values but also by how those values were structurally organized and articulated in ways that were intelligible to Western readers.

Our analysis of the seven virtues revealed that *Meiyo* (名誉; honor) emerged as the psychologically central virtue within *Bushido*'s moral structure based on the MFQ-2-derived CCR scores. In the MDS map constructed from these scores, *Meiyo* was positioned highly on both PCA-interpreted dimensions, indicating that it integrates diverse moral dimensions within *Bushido*'s ethical system. As Nitobe emphasized, *Meiyo*—regarded as the most esteemed virtue—stands for personal dignity and integrity and encourages individuals to avoid shameful behavior (13). Consistent with this, an exploratory CCR analysis using an established integrity scale (36) showed that *Meiyo* exhibited the highest estimated semantic alignment with integrity-related constructs among the seven virtues (see Supplementary information). In addition, in *Bushido*, *Meiyo* is characterized as attainable through the cultivation of patience and self-restraint in suppressing the desire to display one's achievements. Conceptually, *Meiyo* reflects a culturally distinctive moral ideal within Japanese ethical thought, which differs markedly from the Western, pride-based conception of “honor” (37). These findings suggest that *Bushido* rendered Japanese moral ideals intelligible within Western moral frameworks, while preserving the core structure of its indigenous value system.

Our findings suggest that *Bushido* articulates a coherent moral and psychological worldview rooted in less-WEIRD traditions, while exhibiting rhetorical and structural features that render these values intelligible to WEIRD audiences. The present results highlight how classical texts can convey moral concerns in forms that remain interpretable across cultural contexts (3). By applying a theory-driven, computational language analysis grounded in psychology, this study provides empirical support for the view that moral values can function as communicative resources that are shared, interpreted, and rearticulated in different social and cultural settings (3, 15, 38–40). In this sense, *Bushido* illustrates how moral values rooted in less-WEIRD traditions can be preserved and meaningfully conveyed within contexts shaped by WEIRD modes of moral understanding, without being reduced to or replaced by them.

The present findings demonstrate that classical texts, as cultural products, can be analyzed in terms of their latent psychological characteristics as structured systems. By applying CCR and MDS, this study provides a quantitative framework for identifying central and peripheral elements within historically embedded moral structures. In the case of *Bushido*, the results suggest that the seven virtues were organized around *Meiyo* (名誉) as a central moral core, indicating that *Bushido*'s ethical system possessed an internally coherent structure. More broadly, this approach offers an entry point for historical psychological analyses of value systems articulated in cultural texts (22, 41, 42).

Further, these findings offer new perspectives for cultural and moral psychology. *Bushido* represents a text in which moral values rooted in Japanese cultural traditions were organized and articulated in a form that was intelligible to readers from a different cultural background, a feature that may have been associated with its reception in Western contexts. This suggests that understanding moral and cultural values requires attention not only to the *content* of those values but also to *how* they are structured and presented in ways that facilitate interpretation and uptake by others.

While this study showed how CCR enabled a psychological investigation of the Japanese classic *Bushido*, certain limitations remain. First, the present study compared *Bushido* with Christianity-related, Bible-related, and Japanese culture-related documents available in English through Project Gutenberg, and examined Western reception only within the subset of Japanese culture-related documents. This design allowed us to compare texts circulating within Western intellectual contexts on a shared linguistic and computational basis, enabling a precise analysis of both *Bushido*'s moral profile relative to other religious and cultural corpora and, within Japanese cultural texts, the association between moral structure and Western reception. At the same time, this approach does not directly capture pragmatic nuances specific to Japanese-language expression, nor cultural meanings that may be attenuated or transformed through English as a mediating language. Accordingly, the present findings should be interpreted as illustrating how Japanese moral values were articulated in English and understood within Western intellectual contexts, rather than as a direct analysis of moral expression in Japanese-language texts themselves. Second, the WRI used in this study captures indirect indicators of reception, such as publication counts, translations into non-English Western European languages, and citation frequency, which are commonly employed in sociological and bibliometric research. However, these indicators do not directly assess readers' evaluations or interpretive responses. Accordingly, the WRI should be interpreted as a contextual proxy for Western reception rather than as a direct measure of psychological appeal. Third, the CCR approach relies on contemporary psychological constructs as a common metric for quantifying the moral characteristics of texts from different historical and cultural contexts. While this common metric enables systematic comparison, moral concerns and their cultural salience may change over time (43, 44). Therefore, the present analysis should not be interpreted as reconstructing how moral concepts were understood in the historical context in which *Bushido* was written. Relatedly, our findings should not be taken as representing the essence of historical samurai ethics. Rather, they characterize *Bushido* as a modern English-language text that presented Japanese moral ideals to Western readers. Future research should compare *Bushido* with other texts on samurai ethics, including historical Japanese sources and later interpretive writings, by constructing broader comparative corpora and, where necessary, applying translation procedures or multilingual analytic pipelines in a consistent manner.

In conclusion, this study employed an advanced natural language processing technique to quantitatively uncover the moral and psychological structure of *Bushido*. The findings show that *Bushido* expresses moral values rooted in Japanese cultural traditions through a structured moral profile that corresponds to psychologically defined dimensions of moral judgment. The present results demonstrate how a classical less-WEIRD text can render its moral tradition intelligible to WEIRD modes of moral understanding, illustrating how less-WEIRD cultural values can be communicated across cultural boundaries without losing their distinctive meaning.

## Methods

### Document data description

We used text data from 722 documents downloaded from Project Gutenberg (https://www.gutenberg.org/ebooks), including *Bushido* and a large set of Christianity-, Bible-, and Japanese culture-related documents. All documents analyzed in the present study were English-language texts, including English originals and authorized English translations available through Project Gutenberg. Specifically, 225 documents were categorized as Christianity-related and 466 as Bible-related, based on the Library of Congress Classification system commonly used in academic libraries in the United States. The Christianity-related documents were classified under “Philosophy, Psychology, Religion: Christianity,” which covers works on Christianity as a historical, institutional, and cultural tradition, including church history, Christian figures, the Reformation, and related religious writings. Examples include *The Confessions of St. Augustine* and Leo Tolstoy's *The Kingdom of God Is Within You*. The Bible-related documents were categorized under “Philosophy, Psychology, Religion: Christianity: The Bible, The Old and New Testament,” which includes biblical texts and translations as well as Bible-related works such as interpretations, commentary, and Bible stories. Examples include biblical texts and translations such as *The Bible, King James Version* and *Weymouth New Testament in Modern Speech*. Additionally, we included a set of 30 documents thematically related to Japanese culture, selected from the same classification categories as *Bushido*, namely, “Japan—Civilization,” “Japan—Social life and customs,” and “Ethics—Japan.” These texts were labeled as Japanese culture-related documents in our analyses to provide a culturally grounded reference point for interpreting *Bushido*. Examples include *The Book of Tea* and *Tales of Old Japan*. For each document, we used the full textual content, excluding nonessential sections such as Project Gutenberg metadata, translator introductions, and indexes. Accordingly, comparisons of *Bushido* with the Christianity-related, Bible-related, and Japanese culture-related corpora were based on the full text of *Bushido*. By contrast, analyses of the seven virtues were restricted to the virtue-specific chapters: Chapter III for *Gi* (義; rectitude), Chapter IV for *Yu* (勇; courage), Chapter V for *Jin* (仁; benevolence), Chapter VI for *Rei* (礼; politeness), Chapter VII for *Makoto* (誠; veracity), Chapter VIII for *Meiyo* (名誉; honor), and Chapter IX for *Chugi* (忠義; loyalty).

### Data analysis

#### Contextualized construct representations

To examine how moral concerns are reflected in the texts, we employed the CCR method, a technique designed to quantify psychological constructs in naturalistic language (21, 23). In contrast to traditional content coding or dictionary-based word counting, which typically relies on prespecified word lists and can be sensitive to polysemy and context, CCR operationalizes a construct through its theoretically grounded questionnaire items rather than researcher-selected “seed words” (35, 45). CCR also differs from embedding-based dictionary approaches, such as distributed dictionary representation, which represent constructs via a small set of seed words and can be sensitive to the particular words selected and their decontextualized representations (46). By anchoring constructs in psychometrically validated scale items, CCR retains interpretability and top-down flexibility while leveraging the representational power of modern contextual language models. Thus, CCR provides an interpretable, construct-level measure of semantic alignment that complements prior dictionary- and embedding-based approaches.

CCR involves three key steps: first, selecting a psychometrically validated self-report measure; second, embedding both the questionnaire items and target texts using contextualized language models; and third, computing the similarity (e.g. cosine similarity) between each item and segments of the text. The resulting similarity score, referred to as a “CCR loading score,” indicates the degree to which a given construct is represented in the text. Higher scores reflect stronger alignment with the construct of interest. We employed a well-established psychometric instrument to assess moral foundations: the MFQ-2 developed by Atari et al. (29). This instrument was selected for having a large number of items, sufficient coverage of each factor, and balanced item distribution, which are essential for generating stable CCR scores. For further methodological details on CCR, see Supplementary information and Atari et al. (21).

For the CCR analysis, we used Sentence Transformer models to compute semantic sentence similarity, as these models achieve state-of-the-art performance on semantic textual similarity tasks. Specifically, we used the “all-mpnet-base-v2” model, which has been shown to yield the highest performance among Sentence Transformer models available on Hugging Face (https://www.sbert.net/index.html). The analysis was conducted using the “CCR” package in R (ver. 4.4.3) (21). To assess the robustness of our findings, we also computed CCR scores using three additional high-performing general-purpose models and compared the results to evaluate consistency across models (see the Robustness checks section).

CCR is useful in that it enables latent semantic representations in text to be interpreted within a psychological theoretical framework by using questionnaire items as a reference set. At the same time, several assumptions and limitations of this approach have been noted (25). First, CCR estimates depend on the conceptual validity and internal consistency of the questionnaire scales used as anchors. Second, even identical words or sentences may be embedded in different semantic spaces depending on their context or domain (e.g. books vs. social media), which can render the estimated semantic correspondences unstable. In light of these considerations, the present study complemented CCR with a dictionary-based approach grounded in different assumptions using the MFD (34) and examined the validity of the resulting CCR scores through robustness checks (see the Robustness checks section).

#### Comparison of moral profiles across texts

The CCR scores were first computed at the sentence level for each MFQ-2 item across all documents. For comparisons between *Bushido* and the three comparison corpora, these sentence-level scores were then averaged within each document for each moral foundation–item combination, yielding document-level CCR scores. For the analysis of *Bushido*'s seven virtues, by contrast, sentence-level CCR scores were retained so that virtue-specific chapters could be compared within the same text.

We analyzed CCR scores using linear mixed-effects models implemented in the “lmerTest” package in R (47). For comparisons between *Bushido* and the three comparison corpora, document-level CCR scores were analyzed with document type (*Bushido*, Christianity-related, Bible-related, or Japanese culture-related), moral foundation (Authority, Care, Equality, Ingroup, Proportionality, or Purity), and their interaction as fixed effects. Random intercepts were included for individual documents and for MFQ-2 items nested within moral foundations. These random intercepts were included to account for two sources of nonindependence in the data: each document contributed multiple CCR observations across MFQ-2 items, and each MFQ-2 item was applied repeatedly across documents. The random intercept for individual documents accounts for document-specific baseline differences in CCR scores, whereas the random intercept for MFQ-2 items accounts for item-specific baseline differences across documents. Model-fit indices indicated that including both random intercepts improved model fit relative to models including only one of these random-intercept terms (see Table S8). Type III tests with Satterthwaite's approximation for denominator degrees of freedom were used to evaluate the main effects of document type and moral foundation, as well as their interaction. To determine whether *Bushido* differed from each comparison corpus in its overall moral profile, we also fit follow-up pairwise models comparing *Bushido* separately with Christianity-related, Bible-related, and Japanese culture-related corpora. We then estimated marginal means for each document type × moral foundation combination and conducted Dunnett-adjusted planned contrasts comparing *Bushido* with each comparison corpus within each moral foundation.

For the analysis of *Bushido*'s seven virtues, sentence-level CCR scores were analyzed with virtue type (*Gi*, *Yu*, *Jin*, *Rei*, *Makoto*, *Meiyo*, or *Chugi*), moral foundation, and their interaction as fixed effects. Random intercepts were included for MFQ-2 items nested within moral foundations and for individual sentences nested within virtue-specific chapters to account for repeated CCR observations from the same sentence and repeated applications of the same MFQ-2 item across sentences. The random intercept for individual sentences accounts for sentence-specific baseline differences in CCR scores, whereas the random intercept for MFQ-2 items accounts for item-specific baseline differences across sentences. Model-fit indices indicated that including both random intercepts improved model fit relative to models including only one of these random-intercept terms (Table S9). The primary goal of this analysis was to test whether the seven virtues differed in their moral profiles across the six MFQ-2 moral foundations.

We employed MDS to visualize a 2D configuration based on the similarity structure among the seven virtues in *Bushido*. The input for MDS was a dissimilarity matrix calculated using Euclidean distances derived from the 7 × 6 matrix of mean MFQ-2-based CCR scores across *Bushido*'s seven virtues and the six moral foundations. As MDS does not provide interpretable axes by default, PCA was performed on the original CCR scores for the MFQ-2 to identify the primary dimensions of variance in the data. Specifically, we extracted the first two principal components (PC1 and PC2) and compared them to the MDS coordinates to aid the interpretation of the map's underlying structure. To further ensure that the MDS solution's orientation was meaningfully aligned with the psychological constructs, we conducted Procrustes analysis to align the MDS configuration with the PCA-derived dimensions.

#### WRI and moral profile similarity to *Bushido*

To examine the extent to which Japanese culture-related documents, including *Bushido*, have been received in Western contexts and how such reception relates to moral profile similarity to *Bushido*, we constructed a WRI for each document. The WRI was operationalized as a composite index based on indicators commonly used in sociology and bibliometrics (30–33): (i) the number of English-language editions, (ii) the number of translations into five non-English Western European languages, and (iii) citation counts indexed in Google Scholar, restricted to English-language sources. Data on English-language editions and translations were obtained from WorldCat, the world's largest union catalog integrating bibliographic and holdings information from libraries worldwide. For the translation-count component, we counted translations into French, German, Spanish, Italian, and Dutch. All bibliographic and citation data used to construct the WRI were collected in January 2026. Each of the three variables was log-transformed and standardized, and the WRI score was computed as their mean. The three indicators comprising the WRI showed acceptable internal consistency (Cronbach's *α* = 0.78).

Moral profile similarity to *Bushido* was quantified by averaging MFQ-2-based CCR scores across items within each moral foundation for each document and mean-centering scores for each foundation across *Bushido* and the Japanese culture-related documents to account for foundation-specific baseline differences. We then computed the Pearson correlation across the six moral foundations between each document's resulting moral profile and that of *Bushido*. The association between WRI and moral profile similarity was assessed using Spearman's rank-order correlation, excluding *Bushido* itself.

### Ethics statement

This study analyzed publicly available book texts obtained from Project Gutenberg and did not involve human participants, animal subjects, or the collection of personal data. Therefore, institutional review board approval and informed consent were not required.

## Supplementary Material

pgag314_Supplementary_Data

## Data Availability

All shareable data are available on the Open Science Framework repository at https://osf.io/v8sq5/(48). All code is available on the Open Science Framework repository at https://osf.io/v8sq5/ (48).
