# Supplementary material for: The seven virtues of Samurai: Revisiting a classic through computational language analysis
Source: PNAS Nexus. 2026 Sep 17;5(9):pgag314. doi: 10.1093/pnasnexus/pgag314 (PMC13623005; doi:10.1093/pnasnexus/pgag314)
Supplement: Supplementary file 1 [file pgag314_supplementary_data.pdf]

Supplementary Information for

The seven virtues of *Samurai*: Revisiting a classic through computational language analysis

Ryota Takano[1,*] & Minoru Karasawa[2,*]

[1] Department of Cognitive and Psychological Sciences, Graduate School of Informatics, Nagoya University, Nagoya, Japan

[2] Future Value Creation Research Center, Nagoya University, Nagoya, Japan

[*] Correspondence concerning this article should be addressed to Ryota Takano (takano@i.nagoya-u.ac.jp) or Minoru Karasawa (mkarasawa91@gmail.com).

Statistical Analysis

Methodological Overview of Contextualized Construct Representation (CCR)

Contextualized Construct Representation (CCR) is a natural language processing technique designed to quantify the semantic alignment between textual content and psychological constructs represented by validated psychometric scales (1–3). This approach enables a theory-driven, fine-grained analysis of meaning within texts, offering a scalable method for psychological interpretation. In this study, we applied CCR to analyze *Bushido* in comparison with Christianity-, Bible-, and Japanese culture-related texts. A well-established psychological framework was used: the Moral Foundations Questionnaire-2 (MFQ-2) (4). The CCR procedure follows the methodology introduced by Atari *et al.* (1).

As illustrated in Fig. S1, CCR begins by transforming both the target sentence (e.g., from *Bushido*) and the reference item (e.g., from MFQ-2) into high-dimensional semantic vectors using a pretrained language model (e.g., sentence-BERT). Specifically, we used the "all-mpnet-base-v2" model from the Sentence Transformer library, which is known for its strong performance in sentence-level semantic similarity tasks (see Robustness Checks in the main text for comparisons with alternative models). Each sentence is first entered into the model to obtain contextualized word embeddings. These embeddings are then aggregated using mean pooling to generate a fixed-length sentence embedding. The CCR score is computed as the cosine similarity between two vectors, i.e., v representing the embedding of a *Bushido* sentence and u representing that of a questionnaire item:

$$CCR\ score\ = \cos(u, v) = \frac{u \cdot v}{||u||\,||v||}$$

This score reflects the degree of psychological correspondence between the semantic content of the target sentence and the construct represented by the questionnaire item.

In the document-level analysis, we first computed CCR scores between each sentence in a document and each MFQ-2 item. These sentence-level scores were then averaged within each document for each moral foundation–item combination, yielding document-level CCR scores. This aggregation allowed us to compare the overall moral profiles of *Bushido* and the Christianity-related, Bible-related, and Japanese culture-related corpora at the document level.

In contrast, for the virtue-level analysis of *Bushido*, we retained sentence-level CCR scores because each virtue is discussed in a separate chapter. This approach enabled a fine-grained comparison of moral profiles across virtues based on sentence-level variation within *Bushido*. The linear mixed-effects models for the seven virtues were conducted using these sentence-level CCR scores. For visualization and dimensional analyses such as multidimensional scaling (MDS) and principal component analysis (PCA), sentence-level scores were summarized into mean virtue-level profiles across the six MFQ-2 moral foundations.

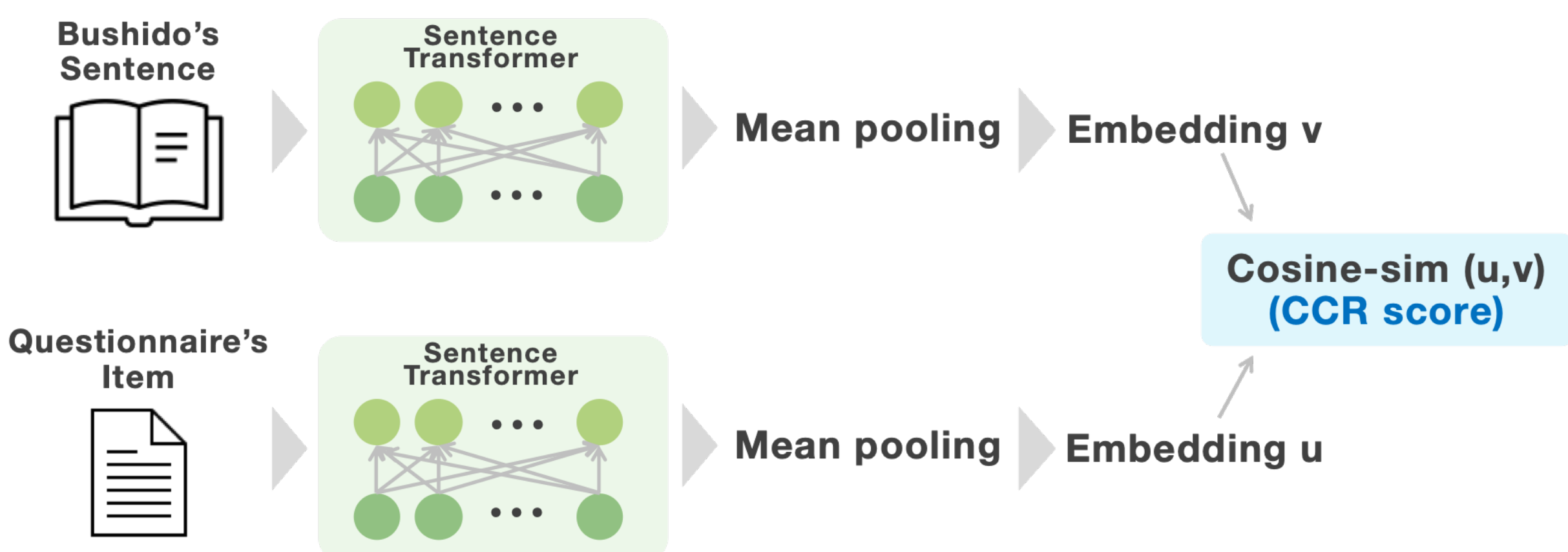

Fig. S1: Schematic pipeline of Contextualized Construct Representation (CCR).
Note. This figure illustrates the process of computing Contextualized Construct Representation (CCR) scores. Each target sentence and each questionnaire item were separately encoded into sentence embeddings using the pretrained Sentence Transformer model and mean pooling. The cosine similarity between the two embeddings represents the CCR score, indicating the degree of semantic alignment between the target sentence and the psychological construct represented by the questionnaire item.

Supplementary Results

Integrity Profiles Across the Seven Virtues of *Bushido*

We conducted an exploratory CCR analysis using an established integrity scale (5). Using the same procedure as in the main MFQ-2 virtue-level analysis, CCR scores were computed for each sentence–item pair within each virtue-specific chapter of *Bushido*. These sentence-level CCR scores were analyzed using a linear mixed-effects model with virtue as a fixed effect and random intercepts for integrity-scale items and individual sentences. The model revealed a significant main effect of virtue on integrity-related CCR scores, $F(6, 457.24) = 3.59$, $p = .002$. Among the seven virtues, *Meiyo* (名誉; honor) exhibited the highest estimated marginal mean (*EMM* = 0.230, 95% CI [0.200, 0.261]). This pattern is consistent with the interpretation of *Meiyo* as a virtue centered on personal dignity and moral integrity.

Table S1. Type III ANOVA results from linear mixed-effects models for MFQ-2-based CCR scores in the full sample and in follow-up pairwise comparisons with *Bushido*.

Comparison	Effect	Num df	Den df	*F* value	*p*
All document types (Full model)	Document type	3	721.1	10.01	< .001
	Foundation	5	37.6	3.45	.012
	Document type × foundation	15	25235.0	79.73	< .001
Bushido vs. Christianity-related documents	Document type	1	225.1	1.94	.165
	Foundation	5	40.3	3.92	.005
	Document type × foundation	5	7875.0	3.04	.010
Bushido vs. Bible-related documents	Document type	1	466.1	3.16	.076
	Foundation	5	42.6	4.16	.004
	Document type × foundation	5	16310.0	4.52	< .001
Bushido vs. Japanese culture-related documents	Document type	1	30.8	4.65	.039
	Foundation	5	43.7	4.59	.002
	Document type × foundation	5	1050.0	3.79	.002

Note. Reported values are Type III tests from linear mixed-effects models. Num df = numerator degrees of freedom; Den df = denominator degrees of freedom. Follow-up pairwise models compared *Bushido* with each comparison corpus separately.

Table S2. Estimated marginal means of MFQ-2-based CCR scores by document type and moral foundation, with Dunnett-adjusted contrasts against *Bushido*.

Foundation	Document type	Estimated marginal mean	Difference from *Bushido*	Dunnett-adjusted *p* value
Authority	*Bushido*	0.177		
	Christianity-related documents	0.124	-0.054	.099
	Bible-related documents	0.105	-0.072	.015
	Japanese culture-related documents	0.098	-0.079	.007
Care	*Bushido*	0.144		
	Christianity-related documents	0.119	-0.025	.633
	Bible-related documents	0.119	-0.025	.632
	Japanese culture-related documents	0.074	-0.069	.023
Equality	*Bushido*	0.090		
	Christianity-related documents	0.062	-0.028	.548
	Bible-related documents	0.048	-0.042	.250
	Japanese culture-related documents	0.051	-0.040	.303
Ingroup	*Bushido*	0.116		
	Christianity-related documents	0.069	-0.047	.179
	Bible-related documents	0.066	-0.050	.139
	Japanese culture-related documents	0.063	-0.053	.114
Proportionality	*Bushido*	0.103		
	Christianity-related documents	0.071	-0.032	.454
	Bible-related documents	0.061	-0.042	.250
	Japanese culture-related documents	0.052	-0.052	.127
Purity	*Bushido*	0.115		
	Christianity-related documents	0.089	-0.027	.590
	Bible-related documents	0.086	-0.029	.524
	Japanese culture-related documents	0.068	-0.047	.181

Note. Estimated marginal means were derived from the linear mixed-effects model reported in the main text. *p* values were adjusted using Dunnett's method for comparisons with *Bushido* within each foundation. Differences are reported as comparison corpus values minus *Bushido* values, so negative values indicate higher scores for *Bushido*.

Table S3. Type III ANOVA results from linear mixed-effects models for MFQ-2-based CCR scores in the contemporaneous-text subset and in follow-up pairwise comparisons with *Bushido*.

Comparison	Effect	Num df	Den df	*F* value	*p*
All document types (Full model)	Document type	3	461.1	5.54	< .001
	Foundation	5	37.3	3.40	.012
	Document type × foundation	15	16135.0	66.50	< .001
Bushido vs. Christianity-related documents	Document type	1	160.2	2.24	.136
	Foundation	5	40.1	3.97	.005
	Document type × foundation	5	5600.0	2.70	.019
Bushido vs. Bible-related documents	Document type	1	274.1	2.64	.105
	Foundation	5	40.7	4.17	.004
	Document type × foundation	5	9590.0	4.61	< .001
Bushido vs. Japanese culture-related documents	Document type	1	27.8	4.59	.041
	Foundation	5	43.9	4.63	.002
	Document type × foundation	5	945.0	3.76	.002

Note. Reported values are Type III tests from linear mixed-effects models. The contemporaneous-text subset included comparison documents authored by individuals whose lifespans overlapped with Inazo Nitobe's (1862–1933). Num df = numerator degrees of freedom; Den df = denominator degrees of freedom. Follow-up pairwise models compared *Bushido* with each comparison corpus separately.

Table S4. Estimated marginal means of MFQ-2-based CCR scores by document type and moral foundation in the contemporaneous-text subset, with Dunnett-adjusted contrasts against *Bushido*.

Foundation	Document type	Estimated Marginal Means	Difference from *Bushido*	Dunnett-adjusted *p* value
Authority	*Bushido*	0.177		
	Christianity-related documents	0.122	-0.055	.081
	Bible-related documents	0.114	-0.063	.037
	Japanese culture-related documents	0.098	-0.080	.006
Care	*Bushido*	0.144		
	Christianity-related documents	0.113	-0.031	.470
	Bible-related documents	0.122	-0.021	.719
	Japanese culture-related documents	0.075	-0.069	.021
Equality	*Bushido*	0.090		
	Christianity-related documents	0.061	-0.029	.515
	Bible-related documents	0.053	-0.037	.340
	Japanese culture-related documents	0.051	-0.039	.301
Ingroup	*Bushido*	0.116		
	Christianity-related documents	0.067	-0.048	.146
	Bible-related documents	0.068	-0.048	.154
	Japanese culture-related documents	0.062	-0.054	.099
Proportionality	*Bushido*	0.103		
	Christianity-related documents	0.069	-0.035	.388
	Bible-related documents	0.066	-0.037	.330
	Japanese culture-related documents	0.052	-0.052	.117
Purity	*Bushido*	0.115		
	Christianity-related documents	0.084	-0.031	.471
	Bible-related documents	0.089	-0.027	.575
	Japanese culture-related documents	0.068	-0.047	.171

Note. This table reports results from the robustness analysis restricted to comparison documents authored by individuals whose lifespans overlapped with Nitobe's (1862–1933). Estimated marginal means were derived from the linear mixed-effects model reported in the main text. *p* values were adjusted using Dunnett's method for comparisons with *Bushido* within each foundation. Differences are reported as comparison corpus values minus *Bushido* values, so negative values indicate higher scores for *Bushido*.

Table S5. Type III ANOVA results from the linear mixed-effects model examining MFQ-2-based CCR scores across *Bushido*'s seven virtues and six moral foundations.

**Effect**	**Num df**	**Den df**	***F* value**	***p***
Virtue type	6	457.2	3.56	.002
Foundation	5	36.0	3.93	.006
Virtue type × foundation	30	15995.0	24.83	< .001

Note. Reported values are Type III tests from the linear mixed-effects model. Num df = numerator degrees of freedom; Den df = denominator degrees of freedom.

Table S6. Mean Contextualized Construct Representation (CCR) scores for the seven virtues in *Bushido* across MFQ-2 foundations

	Foundation					
**Virtue**	Authority	Care	Equality	Ingroup	Proportionality	Purity
Gi (義)	0.21<br>(0.09)	0.15<br>(0.11)	0.10<br>(0.09)	0.13<br>(0.10)	0.12<br>(0.08)	0.13<br>(0.09)
Yu (勇)	0.16<br>(0.11)	0.17<br>(0.12)	0.05<br>(0.10)	0.13<br>(0.09)	0.09<br>(0.09)	0.10<br>(0.09)
Jin (仁)	0.20<br>(0.10)	0.19<br>(0.13)	0.11<br>(0.11)	0.14<br>(0.08)	0.12<br>(0.09)	0.11<br>(0.09)
Rei (礼)	0.21<br>(0.12)	0.15<br>(0.11)	0.12<br>(0.09)	0.10<br>(0.07)	0.12<br>(0.09)	0.15<br>(0.10)
Makoto (誠)	0.18<br>(0.10)	0.12<br>(0.11)	0.11<br>(0.10)	0.11<br>(0.07)	0.12<br>(0.09)	0.10<br>(0.09)
Meiyo (名誉)	0.22<br>(0.11)	0.20<br>(0.11)	0.10<br>(0.09)	0.15<br>(0.08)	0.14<br>(0.09)	0.14<br>(0.10)
Chugi (忠義)	0.20<br>(0.11)	0.16<br>(0.10)	0.09<br>(0.09)	0.15<br>(0.10)	0.11<br>(0.08)	0.11<br>(0.10)

Note. Values represent mean CCR scores, and the numbers in parentheses indicate standard deviations. CCR scores were computed for all item-sentence pairs within each chapter and MFQ-2 foundation.

Table S7. Mean Contextualized Construct Representation (CCR) scores for chapters other than the seven virtues in *Bushido* across MFQ-2 Foundations

	Foundation					
**Chapter**	Authority	Care	Equality	Ingroup	Proportionality	Purity
Bushido As an Ethical System	0.20 (0.04)	0.12 (0.08)	0.09 (0.04)	0.12 (0.03)	0.10 (0.05)	0.11 (0.06)
Sources of Bushido	0.19 (0.03)	0.13 (0.08)	0.10 (0.04)	0.10 (0.03)	0.09 (0.05)	0.11 (0.06)
The Education and Training of a Samurai	0.18 (0.04)	0.11 (0.08)	0.12 (0.03)	0.10 (0.03)	0.11 (0.04)	0.11 (0.05)
Self-Control	0.17 (0.04)	0.19 (0.05)	0.09 (0.04)	0.12 (0.03)	0.09 (0.04)	0.15 (0.03)
The Institutions of Suicide and Redress	0.13 (0.03)	0.15 (0.05)	0.05 (0.05)	0.09 (0.02)	0.08 (0.04)	0.11 (0.05)
The Sword, The Soul of the Samurai	0.13 (0.02)	0.12 (0.05)	0.04 (0.04)	0.10 (0.02)	0.07 (0.04)	0.11 (0.04)
The Training and Position of Woman	0.19 (0.04)	0.14 (0.06)	0.11 (0.04)	0.11 (0.02)	0.12 (0.05)	0.13 (0.07)
The Influence of Bushido	0.18 (0.04)	0.13 (0.07)	0.08 (0.05)	0.12 (0.01)	0.11 (0.05)	0.10 (0.06)
Is Bushido Still Alive?	0.18 (0.03)	0.14 (0.06)	0.10 (0.04)	0.13 (0.01)	0.11 (0.05)	0.10 (0.05)
The Future Of Bushido	0.19 (0.03)	0.13 (0.07)	0.10 (0.04)	0.13 (0.02)	0.10 (0.04)	0.11 (0.06)

Note. Values represent mean CCR scores, and the numbers in parentheses indicate standard deviations. CCR scores were computed for all item-sentence pairs within each chapter and MFQ-2 foundation.

Table S8. Model comparison results for random-intercept structures in linear mixed-effects analyses comparing *Bushido* with the Christianity-related, Bible-related, and Japanese culture-related corpora.

Models	AIC	BIC	Deviance	Log-likelihood
null model	-127311	-127278	-127319	63659
model 1	-128478	-128258	-128532	64266
model 2	-106266	-106054	-106318	53159
model 3	-94223	-94010	-94275	47137

Note. AIC: Akaike information criterion, BIC: Bayesian information criterion, null model: intercept-only with no predictors, model 1: two random intercepts for questionnaire items and individual documents, model 2: one random intercept for the questionnaire item; model 3: one random intercept for individual documents.

Table S9 Model comparison results for random-intercept structures in linear mixed-effects analyses of *Bushido*'s seven virtue chapters.

Models	AIC	BIC	Deviance	Log-likelihood
null model	-40314	-40283	-40322	20161
model 1	-40996	-40649	-41086	20543
model 2	-35186	-34847	-35274	17637
model 3	-33452	-33112	-33540	16770

Note. AIC: Akaike information criterion, BIC: Bayesian information criterion, null model: intercept-only with no predictors, model 1: two random intercepts for questionnaire items and individual sentences, model 2: one random intercept for questionnaire item; model 3: one random intercept for individual sentences.

References.

1. M. Atari, A. Omrani, M. Dehghani, Contextualized Construct Representation: Leveraging psychometric scales to advance theory-driven text analysis. *PsyArxiv* (2023).

2. M. E. W. Varnum, N. Baumard, M. Atari, K. Gray, Large Language Models based on historical text could offer informative tools for behavioral science. *Proc. Natl. Acad. Sci. U. S. A.* **121**, e2407639121 (2024).

3. Y. Chen, S. Li, Y. Li, M. Atari, Surveying the Dead Minds: Historical-Psychological Text Analysis with Contextualized Construct Representation (CCR) for Classical Chinese in *Proceedings of the 2024 Conference on Empirical Methods in Natural Language Processing*, Y. Al-Onaizan, M. Bansal, Y.-N. Chen, Eds. (Association for Computational Linguistics, 2024), pp. 2597–2615.

4. M. Atari, *et al.*, Morality beyond the WEIRD: How the nomological network of morality varies across cultures. *J. Pers. Soc. Psychol.* **125**, 1157–1188 (2023).

5. B. R. Schlenker, Integrity and character: Implications of principled and expedient ethical ideologies. *J. Soc. Clin. Psychol.* **27**, 1078–1125 (2008).
